# Marine-Derived Xanthone from 2010 to 2021: Isolation, Bioactivities and Total Synthesis

**DOI:** 10.3390/md20060347

**Published:** 2022-05-25

**Authors:** Ana C. S. Veríssimo, Diana C. G. A. Pinto, Artur M. S. Silva

**Affiliations:** LAQV-REQUIMTE, Department of Chemistry, University of Aveiro, 3810-193 Aveiro, Portugal; carolinaana@ua.pt (A.C.S.V.); diana@ua.pt (D.C.G.A.P.)

**Keywords:** xanthones, marine-derived xanthones, bioactive xanthones, total synthesis

## Abstract

Marine life has proved to be an invaluable source of new compounds with significant bioactivities, such as xanthones. This review summarizes the advances made in the study of marine-derived xanthones from 2010 to 2021, from isolation towards synthesis, highlighting their biological activities. Most of these compounds were isolated from marine-derived fungi, found in marine sediments, and associated with other aquatic organisms (sponge and jellyfish). Once isolated, xanthones have been assessed for different bioactivities, such as antibacterial, antifungal, and cytotoxic properties. In the latter case, promising results have been demonstrated. Considering the significant bioactivities showed by xanthones, efforts have been made to synthesize these compounds, like yicathins B and C and the secalonic acid D, through total synthesis.

## 1. Introduction

**Figure 1:**
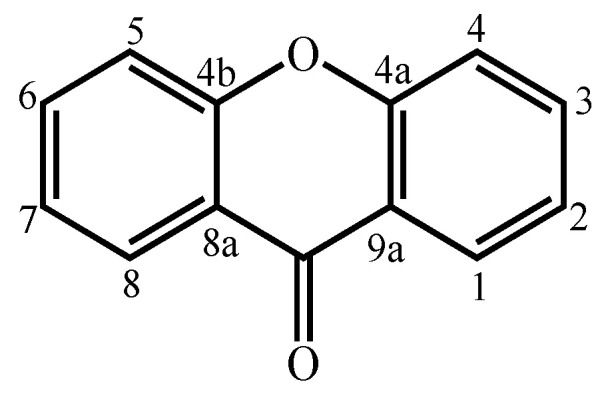
Xanthone (9*H*-xanthen-9-one) core structure.

Xanthones are considered polyketide derivatives due to their biosynthetic precursor. They are aromatic oxygenated heterocyclic compounds with a dibenzo-γ-pyrone scaffold, known as 9*H*-xanthen-9-one (Figure 1) [1]. This molecule can accommodate various substituents at different positions [2], making xanthones recognized as privileged scaffolds in searching for new drugs [1,3].

Xanthones are widely distributed in nature in higher plants, lichens, and fungi from terrestrial origins [4,5,6,7,8,9]. In addition, the marine environment, the least explored area of this planet, has also proven to be an invaluable source [10,11]. Many xanthones have been isolated from marine-derived fungi [10,11,12,13,14,15,16,17,18,19,20,21,22,23,24,25,26,27,28,29,30,31,32,33,34,35,36,37,38,39,40], which can be found in marine sediments and associated with other marine organisms [12,13,14,15,16,17,18,19,20,21,22,23,24,25,26,27,28,29,30,31,32,33,34,35,36,37,38,39,40]. Marine environments, such as temperature, salinity, and pressure, to which marine organisms are subject, sometimes force them to develop unique defenses against the conditions in which they live [41,42]. These unique defenses can lead to the biosynthesis of new secondary metabolites, different from those synthesized by terrestrial sources [41]. The chemically different/unique structures allow xanthones to have important biological activities, such as cytotoxic [12,15,16,17,21,22,23,27,32,37,43], antibacterial [12,16,17,18,19,20,22,26,29,30,35,36,39], and antifungal [20,22,33,40] activities, giving them great potential as natural products with medicinal value [1].

Therefore, the chemical uniqueness of marine-derived xanthones and the significant bioactivities support the idea that the marine environment could be a valuable source of new hits, leads, and drugs [44]. However, access to some marine natural products may be difficult, and in some cases, isolation may yield small amounts of compounds [45]. Different techniques have been applied to increase the availability of the new natural compounds, which can be produced by bacteria or yeasts, or through a chemical approach, such as laboratory synthesis [45]. Synthetic routes can help to overcome supply problems, but it is also very important since it allows the preparation of structures with different substitution patterns relative to those provided by nature, which rise to the opportunity to generate new bioactive agents [46,47,48]. Bioinspiration is very useful in medicinal chemistry as it allows the selection of molecular structures to be used as scaffolds and the strategy to follow for molecular transformations [2].

This review highlights the marine xanthones isolated from 2010 to 2021, emphasizing the ones isolated for the first time in this period and highlighting the most prominent bioactivities presented by the isolated compounds. Finally, the synthetic pathways used for the total synthesis of essential xanthones, such as yicathins B, C, and secalonic acid D, are presented.

## 2. New Marine Xanthones Isolated since 2010

Under the period covered by this review, several studies were carried out to identify biologically active marine compounds, of which the xanthones stand out. Around 100 xanthones were isolated, from which 51 are considered new natural compounds.

Nowadays, the isolation of xanthone derivatives from marine sources usually involves fermentation to increase the compound’s final amount. Fermentation involves putting the fungi in a culture broth, which comprises all the nutrients, such as glucose, iron phosphate, calcium carbonate, malt extract, and controlled pH [21,26], necessary for its growth. The duration of fermentation can be variable but usually involve several days, at least 14 days and the highest 45 days [14,17]. Then, the cultured broth is filtered and extracted, most often, with EtOAc. This extract is subjected to a vacuum liquid chromatography over a silica gel column [15,16,21,23], flash chromatography [19] or, most commonly, to normal column chromatography (CC) [14,17,18,20,22], originating different fractions, which can be subjected to analysis by HPLC or TLC, obtaining sub-fractions, or be directly purified to obtain pure compounds. Most of the xanthones herein referred to were obtained after semi-preparative HPLC purifications of these sub-fractions. Naturally, the eluents and gradients used differ depending on the extracts/fractions being analyzed. The most common eluents used are organic solvents mixtures, such as *n*-hexane/CH_2_Cl_2_/MeOH [16], *n*-hexane/EtOAc [15], CH_2_Cl_2_/MeOH [15,18,20,21,23], gradient of petroleum ether/EtOAc [14,17,18], but mixtures of H_2_O/MeOH can also be used successfully [19].

**Figure 2:**
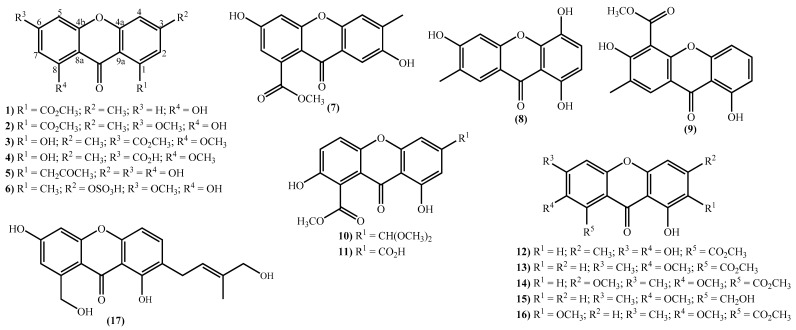
Marine xanthones with simple structures isolated for the first time from 2010 to 2021.

The new xanthone derivatives isolated include simple ones (Figure 2), mainly isolated from marine-derived fungi found in marine sediments [15,18,20,31,32,33,34,35,36,46,47,48,49]. These xanthones are usually highly substituted, with different substituent patterns being the substituents found to be essential for four types; hydroxy, methoxy, methyl, and methoxycarbonyl groups. It seems that the methoxy group is not common in bioactive marine xanthones [50], but yicathins B (**3**) and C (**4**) (Figure 2), which were recently prepared by total synthesis [51], present such a group at C-8. Other examples include xanthones bearing a 6-methoxy group (**2**) and (**6**) and 7-methoxy group (**13**) and (**15**) (Figure 2). On the other hand, marine xanthones (**14**) and (**16**) present two methoxy groups, respectively, at C-3 and C-7 and at C-2 and C-7 (Figure 2).

**Figure 3:**
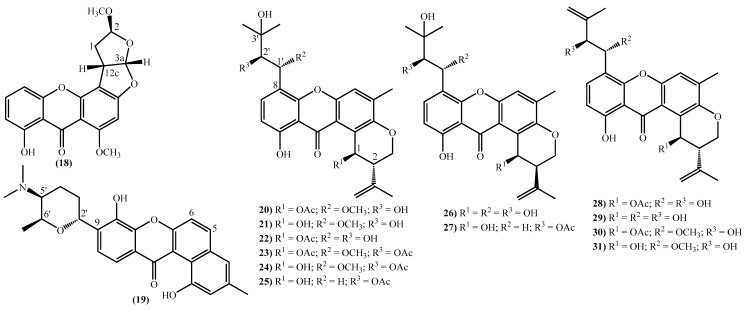

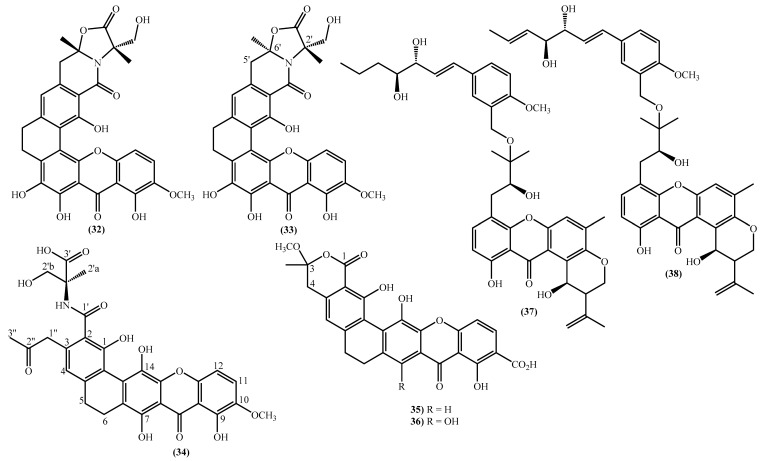
Marine xanthones with more complex structures isolated for the first time from 2010 to 2021.

The more complex xanthones isolated during the covered period are depicted in Figure 3 and present the xanthone skeleton fused with other rings. The most interesting examples include (2*S*,3a*S*,12c*S*)-8-hydroxy-2,6-dimethoxy-1,2,3a,12c-tetrahydro-7*H*-furo [3’,2’:4,5] furo [2,3-*c*] xanthen-7-one (**18**), isolated from the marine fungus *Aspergillus versicolor* and named oxisterigmatocystin D due to its similarity with the known oxisterigmatocystin C [37]. Although the similarities with oxisterigmatocystin C and other isomers were previously isolated, the authors established the stereogenic centers’ configurations and demonstrated that oxisterigmatocystin D is a new natural compound [37]. Compound (**19**), named monacyclione G, is another example isolated from *Streptomyces* sp. HDN15129 [21]. Its stereochemistry was confirmed by the NMR data mainly through the correlations found in HMBC [21] and was established as depicted in Figure 3. 

The most prevalent nucleus in these new marine xanthones is the 11-hydroxy-5-methyl-2-(prop-1-en-2-yl)-2,3-dihydropyrano [3,2-*a*]xanthen-12(1*H*)-one, found in fourteen of the twenty-one new derivatives (Figure 3). Twelve marine xanthones with this core were isolated from the fungus *Aspergillus* sp. ZA-01, respectively, aspergixanthones A-K (**20**–**24** and **26**–**31**) and the 15-acetyltajixanthone hydrate (**25**) (Figure 3) [14,17]. Varioxiranols F (**37**) and G (**38**) (Figure 3) were isolated from the *Emericella variecolor*, a fungus associated with a *Cinachyrella* sp. Sponge [28].

The aspergixanthones (**20**–**24**, **26** and **27**) and the 15-acetyltajixanthone hydrate (**25**) have a 3-hydroxy-3-methylbutyl moiety at C-8, whereas the remaining aspergixanthones (**28**–**31**) have at the same position a 3-methylbut-3-en-1-yl substituent (Figure 3), in both cases, hydroxy, methoxy or acetyloxy groups at C-1′ and C-2′ are also present (Figure 3).

The last examples are xanthone derivatives bearing a 1,7,8,10-tetrahydroxy-11-methoxy-5,6-dihydro-9*H*-naphtho [2,1-*c*]xanthen-9-one (**32**, **33**) and a 1,9,14-trihydroxy-5,6-dihydro-8*H*-naphtho [2,1-*b*]xanthen-8-one nucleus (**34**–**36**) (Figure 3). Buanmycin (**34**) was extracted from a marine *Streptomyces cyaneus* [52], and the remaining examples, citreamicin θ A (**32**), citreamicin θ B (**33**), citreaglycon A (**36**), and dehydrocitreaglycon A (**35**), were isolated from *Streptomyces caelestis* [19].

**Figure 4:**
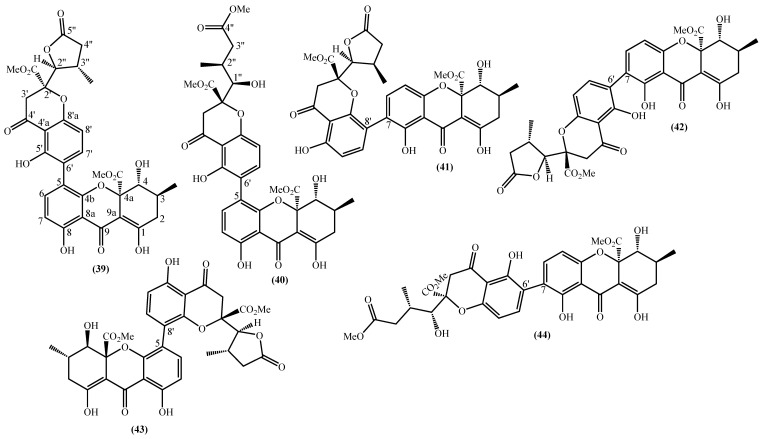

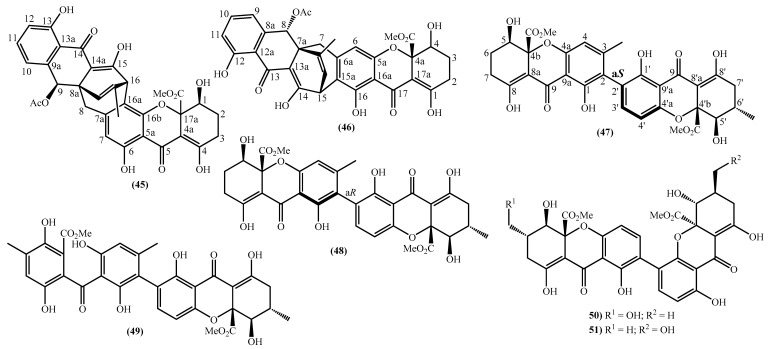
Marine tetrahydroxanthones isolated for the first time from 2010 to 2021.

In the last years, complex tetrahydroxanthone derivatives were also isolated (Figure 4) [23,26,27,40]. Compounds (**39**–**44)**, named versixanthones A-F, were isolated from the fungus *Aspergillus versicolor* HDN1009 [27]. Compounds (**45**, **46**) were isolated from the fungus *Engyodontium* *album* strain LF069 [26] and the *Tritirachium* sp. SpB081112MEf2 a marine sponge fungus [40]. Finally, compounds (**47**–**51**) were also isolated from the fungus *Aspergillus* *versicolor* HDN1009 [23].

Versixanthones A–F have a methyl (3*R*,4*S*,4a*S*)-1,4,8-trihydroxy-3-methyl-9-oxo-2,3,4,9-tetrahydro-4a*H*-xanthene-4a-carboxylate moiety linked to a methyl (2′*R*,2″*S*,3″*S*)-5-hydroxy-2-(3-methyl-5-oxotetrahydrofuran-2-yl)-4-chromanone-2-carboxylate (**39**, **41**–**43**) and methyl (2′*R*,1″*R*,2″*S*)-5-hydroxy-2-(1-hydroxy-4-methoxy-2-methyl-4-oxobutyl)-4-chromanone-2-carboxylate (**40**, **44**). The main differences are the linkage between the two moieties since the authors establish the stereocenter configurations as equal in all isomers (Figure 4) [27].

Compounds (**45**, **46**) represent two diastereomers fully characterized by Wu et al. in 2016 [26], although the isomers JBIR-99 (**45a**) and JBIR-97/98 (**46a**) were previously isolated by Ueda et al. in 2010 [40]. The absolute configuration of these isomers and the engyodontochone A (**46b**) and B (**45b**) was established using NMR experiments and is depicted in Figure 4 [26].

Our final examples are the dimeric tetrahydroxanthones (**47**–**51**) isolated from the fungus *Aspergillus* *versicolor* HDN1009 and named versixanthones G-K (Figure 4) [23]. The structure depicted in Figure 4, including the absolute configuration, was established using NMR experiments.

## 3. Bioactivities Presented by Marine Xanthones

Recognizing the drug potential of xanthone derivatives, several new isolated members were subjected to biological evaluations, in some cases with exciting results. The results are presented and critically discussed [1,2,3]. Although we could find some antifungal activity evaluations, their cytotoxicity and antibacterial potential are the compounds’ most common assessments. For example, compound (**1**) isolated from mangrove fungi, strains nº K38 and E33 [33], is reported to present inhibitory activity of the fungi *Gloeasporium musae*, *Blumeria graminearum*, *Fusarium oxysporum*, *Peronophthora cichoralearum*, and *Colletotrichum glocosporioides* [33]. In particular, the fungi *Gloeasporium musae* (rate of inhibition 53%) and *Peronophthora cichoralearum* (rate of inhibition 48%); however, the authors reported the use of a negative control but not a positive one, so it is difficult to establish the compound potential [33]. Compounds (**2**–**4**), isolated from *Aspergillus wentii* pt-1 found in the marine red alga *Gymnogongrus flabelliformi* and named yicathin A-C, were also evaluated for their ability to inhibit the fungi *Colletotrichum lagenarium, Fusarium oxysporum,* as well as the bacteria *Escherichia coli* and *Staphylococcus aureus*. Unfortunately, the data are incomplete, which limits conclusions regarding the potential of compounds (**2**-**4**) [48]. Nevertheless, it seems that these results motivated the total synthesis of yicathins B (**3**) and C (**4**) [51], which we will discuss in the next section.

The derivatives (**45**, **46**) were evaluated for antifungal activity against *Candida albicans* and *Trichophyton rubrum* [26]. Compounds (**45a**), (**45b**), (**46a**), and (**46b**) demonstrated activity against *T. rubrum* with IC50 values (**45a** 5.3 ± 1.0 μM; **45b** 6.0 ± 1.7 μM; **46a** 4.1 ± 0.8 μM; **46b** 4.3 ± 0.9 μM), which were higher than the positive control (clotrimazol IC_50_ 0.16 ± 0.03 μM) [26]. Compounds (**45a**), (**45b**), (**46a**), and (**46b**) were also evaluated for antibacterial activity against clinically relevant bacteria, *Staphylococcus epidermidis, Staphylococcus aureus*, and *Propionibacterium acnes.* In this case, the results obtained with *S. epidermidis* and *S. aureus* are, for all compounds, lower than the positive control (Table 1) and indicate some drug potential. Furthermore, these compounds (**45a**, **45b**, **46a**, and **46b**)) also inhibited the growth of mouse fibroblast cell line NIH 3T3 with IC_50_ similar to the positive control (Table 1) [26]. Some of these compounds were also evaluated against human cervical carcinoma HeLa cells and human malignant pleural mesothelioma ACC-MESO-1. Unfortunately, the reported values do not have an associated error, and the IC_50_ value of the positive control is not reported [40].

Compound (**8**), 1,4,7-trihydroxy-6-methylxanthone, isolated from marine fungus *Talaromyces islandicus* EN-501, which is found in red alga *Laurencia okamurai,* is another example that was evaluated against several bacterial strains (Table 1) [35]. The obtained values suggest some activity, although much higher than the positive control; moreover, the compound (**8**) antioxidant was also assessed. A potent ability to scavenge the DPPH and ABTS radicals was observed, respectively, IC_50_ 6.92 µg/mL and 2.35 µg/mL. In this case, the values are lower than the positive control, respectively, BHT (IC_50_ 16.27 µg/mL) and ascorbic acid (IC_50_ 3.01 µg/mL), but we should highlight that the authors do not report the value error indicating that only one analysis was performed [35].

The aspergixanthone derivatives (**20**–**24**, **26**, **28**, **29**), isolated from marine-derived *Aspergillus* sp. ZA-01 were evaluated for their antibacterial and cytotoxic activities (Table 1) [17]; unfortunately, there is no evidence that replicates were performed, and the positive control used in the antibacterial activity result was not reported. So, although some derivatives show an interesting ability to inhibit some of the tested cell lines, it is clear that additional studies are essential. The other aspergixanthone derivatives (**27**, **30**, **31**) were evaluated against the three *Vibrio* species, and again it seems that the authors performed just one analysis; nevertheless, the positive control result was reported, which allows some comparisons [14].

The compounds (**32**, **33**, **35**, **36**) were isolated from marine-derived *Streptomyces* sp. and were tested against *Bacillus subtillis* 769, *Staphylococcus haemolyticus* UST950701-004, *Staphylococcus aureus* UST950701-005, and *Staphylococcus aureus* ATCC 43300. The diastereomers (**32**, **33**) showed similar antibacterial activity against these four strains. Compounds (**35**, **36**) also displayed similar biological activity, although compound (**36**) revealed no activity against *S. aureus* ATCC 43300. In general, the values are higher than the positive control penicillin G but similar to the streptomycin value (Table 1) [19]. Therefore, compounds (**32**, **33**) exhibited much stronger antibacterial activity than compounds (**35**, **36**), suggesting that the presence of the five-membered nitrogen heterocycle is essential for the activity. These compounds were also tested for their cytotoxicity against HeLa cells, and again, compounds (**32**, **33**) show significant cytotoxic activity, much higher than the positive control, whereas compounds (**35**, **36**) did not demonstrate the capacity to inhibit the cancer cells in lower concentrations (Table 1) [19].

Our final example of compounds tested for their antibacterial activity is buanmycin (**34**), which was tested against Gram-positive and Gram-negative bacteria (Table 1) [52]. The results revealed that buanmycin (**34**) showed strong inhibition of *S. enterica*, compared with the positive control (Table 1), indicating its drug potential to treat salmonellosis. Additionally, buanmycin (**34**) exhibited potent cytotoxicity against colorectal carcinoma cells (HCT-116), breast cancer cells (MDA-MB231), and gastric carcinoma cells (SNU-638) with IC_50_ values, lower or similar to the ones displayed by the positive control (Table 1) [52].

Regarding cytotoxicity evaluations, compound (**7**) displayed interesting inhibitory values for both Hep-2 (human epidermoid cancer cell line) and HepG2 (human liver cancer cell line) cells (Table 1). However, the authors do not report the positive control result, nor do they report any statistical analysis [34].

The in vitro cytotoxic effects of (**39**–**44**) isolated from the mangrove-derived fungus *Aspergillus versicolor* were evaluated against a cancer cell line panel (HL-60, K562, A549, H1975, 803, HO-8910, and HCT-116). Compounds (**39**–**41**) displayed selective, potent cytotoxicity against HL-60 and K562, while (**42**–**44**) exhibited extensive cytotoxicity against all seven cancer lines, with IC_50_′s ranging from 0.7 to 14.0 μM (Table 1) [27]. Again, we regret highlighting that no statistical data are reported, although some of the displayed values could be comparable to the positive control IC_50_ [27].

From the mangrove-derived fungus *Aspergillus versicolor,* five new dimeric xanthones (**47**–**51**) were also isolated and assessed for their cytotoxic activity against different human cell lines (HL-60, K562, A549, H1975, MGC803, HEK293, HO-8910, HCT-116) [23]. Although no statistical data, nor the IC_50_ value for a positive control were reported, it seems that these compounds exhibited cytotoxicity at different levels (Table 1). Nevertheless, some extra experiments were performed to disclose the compounds’ mechanism of action, and (**47**, **48**, **51**) displayed topo I inhibition activity. The compound (**51**) showed topo I inhibitory activity in a concentration-dependent manner. This study also showed that (**47**) inhibited topo I via trapping the topo I-DNA complex, arresting the cell cycle at the G2/M phase and inducing necrosis in cancer cells [23].

**Figure 5:**
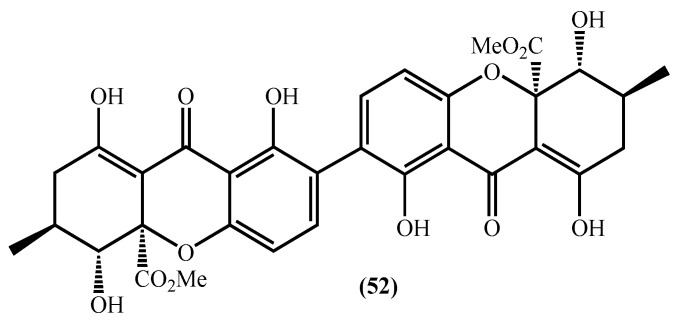
Secalonic acid D structure.

It should be emphasized, in this section, that our aim was to report and discuss the new marine xanthone derivatives found during the period covered by this revision; however, several known marine xanthones were studied and displayed biological activities [53,54,55,56,57,58,59,60,61]. From all those, it is imperative that we highlight secalonic acid D (SAD) (**52**) (Figure 5). This compound was isolated from several marine sources, such as marine-derived *Penicillium oxalicum*, marine sponge-derived fungus *Aspergillus* sp. SCSIO XWS03F03, the marine-derived fungus *Aspergillus versicolor* HDN1009, and marine lichen-derived fungus *Gliocladium* sp. T31 [27,39,43,56,57,62]. However, this compound was isolated for the first time in 1969 from *Penicillium oxalicum* [59]. Before 2010 some studies on the antitumor effects such as leukemia [60] and bladder carcinoma [61] were carried out but never tested for pituitary adenoma. Thus, Liao et al. investigated the anti-pituitary adenoma effect of SAD (**52**) [63], and the authors concluded that the compound in question inhibits the proliferation of pituitary adenoma cells in a time and dose-dependent manner. The cytotoxic effect was mainly due to apoptosis. SAD (**52**) causes G1/S phase arrest and induces apoptosis as indicated by the activation of caspases. Furthermore, the authors also found that SAD (**52**) suppresses the growth hormone (GH) expression levels in GH3 cells, without changing the expression of GH mRNA [63].

Different studies have shown that SAD (**52**) exhibited considerable inhibitory activity on DNA topo I [43,56]. In fact, SAD (**52**) displays a significant inhibition of topo I in a dose-dependent manner and although SAD (**52**) inhibits the binding of topo I to DNA, it does not induce the formation of topo I-DNA covalent complexes. The data explain that SAD (**52**) is a DNA topo I inhibitor rather than a poison-like prototypic DNA topo I poison camptothecin (CPT) [56].

SAD (**52**) was tested against HL-60, K562, A549, H1974, 803, HO-8910, and HCT-116 cell lines presenting interesting results [27]. Moreover, it exerted potent cytotoxicity against various MDR cells and several human lung cancer cells (KB, KBv200, MCF-7, MCF-7/Adr, Kb-3-1, C-A120, S1, S1-M1-80, A549, and GLC-82) (Table 1) [56]. The authors proposed that the SAD (**52**) mechanism of action consists of the ability to down-regulate the expression of ABCG2 and decrease the percentage of SP cells in lung cancer cells due to the induction of ABCG2 degradation by calpain 1 activation [56]. SAD (**52**) showed cytotoxic activity on the human pancreatic carcinoma PANC-1 cells adapted to glucose-starved conditions with an IC_50_ value of 0.6 µM [64]. In addition, SAD (**52**) was also tested for antimicrobial activity, having shown moderate activity against *Staphyloccocus aureus* and *Mycobacterium tuberculosis* [39]. Given its full potential, its total synthesis has been tested in the laboratory and will be addressed later in this review [64].

## 4. Xanthones Obtained by Total Synthesis

Some of the xanthones reported in this review have shown considerable interest on the part of researchers due to their bioactive properties, such as secalonic acid D (**52**) and yicathins B (**3**) and C (**4**), thus showing potential to be promising leads to drug development. However, since these compounds are isolated from marine sources, thus not allowing large amounts, the possibilities of synthesizing them in the laboratory have been explored. The pathways for the total synthase of yicathins B (**3**) and C (**4**) and secalonic acid D (**52**) are described below.

### 4.1. Total Synthesis of Secalonic Acid D

**Scheme 1:**
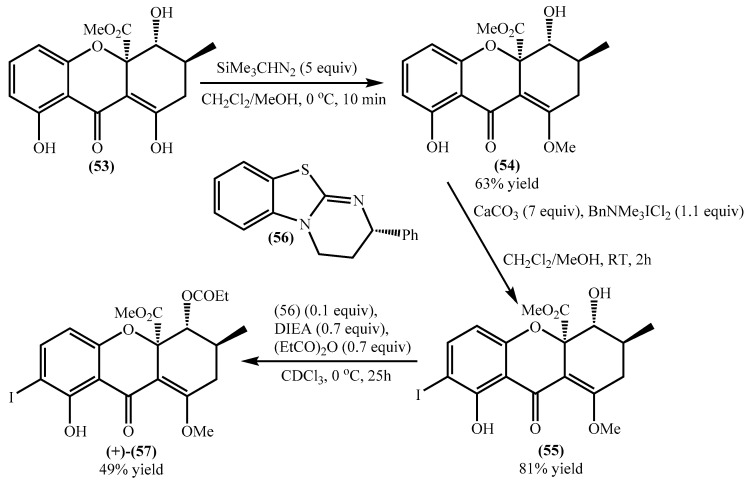
*ortho*-Iodation of blennolide B (**53**).

Qin et al. developed a concise methodology to obtain natural tetrahydroxanthones [65], and during their study blennolide B (**53**) was obtained with an overall yield of 52%. Later on, the same research group used this compound for the secalonic acid D total synthesis [64]. First, it was essential to develop a selective methodology for the *o*-iodination, and the authors used several compounds as models and applied the methodology to a racemic blennolide B (**53**), obtaining the 7-iodo derivative in a very low overall yield (25%) (Scheme 1) [64]. The main problems are related to by-products; for example, during the methylation to obtain compound (**54**) and due to the tautomeric equilibria, another isomer was obtained. Next, the authors obtained the racemic 7-iodoblennolide B (**55**) in excellent yield, and the acylative kinetic resolution afforded both the (−)-7-iodoblennolide B (**55**) and the desired compound (+)-(**57**) in moderate yields, respectively, 48% and 49% [64].

**Scheme 2:**
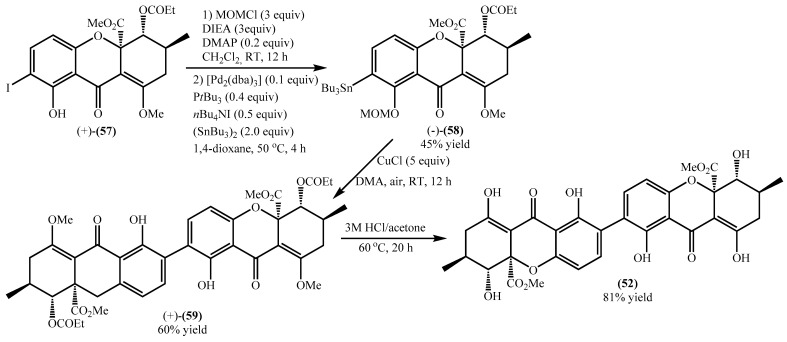
Synthesis of secalonic acid D (**52**).

Encouraged by previous studies and having in hand the desired starting material, the authors performed the synthesis of the secalonic acid D (**52**), which was obtained with a low overall yield of 23% (Scheme 2) [64]. First, the 8-OH was protected with MOM giving an 81% yield. The stannane derivative (−)-(**58**) was made with a 56% yield, and the overall yield of (−)-(**58**) was 45% (Scheme 2). Copper-mediated oxidative coupling provided the dimeric product (+)-(**59**) in good yield, which by an acidic treatment underwent the deprotection and afforded the secalonic acid D (**52**) in excellent yield (Scheme 2). The proposed methodology allows the synthesis of the desired secalonic acid D (**52**) in several steps that, if analyzed separately, gave good to excellent yields but a very low overall yield (10%).

### 4.2. Total Synthesis of Yicathins B and C and Analogues

**Scheme 3:**
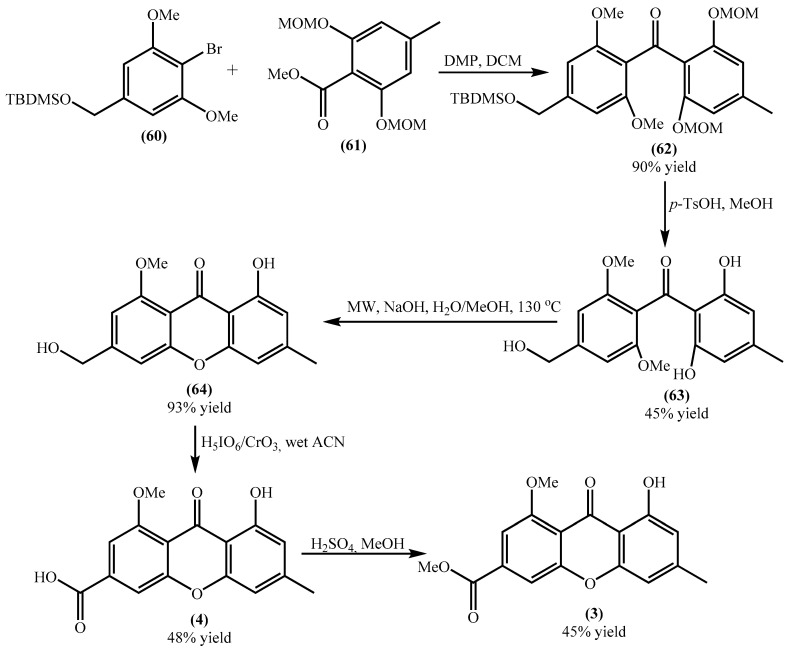
Synthesis of yicathins B (**3**) and C (**4**).

Yicathins B (**3**) and C (**4**) are simple xanthones prevalent in terrestrial plants, besides their occurrence in marine organisms. Due to the medicinal significance of xanthones, they can be considered interesting building blocks for the synthesis of new bioactive compounds. So their synthesis seems to be of utmost importance and was recently achieved (Scheme 3) [51]. The first steps consist of synthesizing the building blocks (**60**) and (**61**), obtained through simple procedures using commercially available reagents, in excellent to good yields, respectively, 87% and 55%.

Acyl substitution of the benzoate (**61**) by the aryllithium intermediate, prepared *in situ* from (**60**), yielded the benzophenone (**62**) in excellent yield. This benzophenone is afterward deprotected, under acidic conditions, to give benzophenone (**63**) in moderate yield (Scheme 3) [51]. Benzophenone (**63**) undergoes an intramolecular nucleophilic aromatic substitution under microwave irradiation affording the xanthone (**64**) in excellent yield. Yicathin C (**4**) was obtained by oxidation with a modified Jones reagent in moderate yield. Yicathin B (**3**) was obtained by Fischer esterification of the carboxylic group in moderate yield (Scheme 3. Considering the overall yield, yicathin C (**4**) and yicathin B (**3**) are obtained, respectively, in 10% and 4%, which are very low yields. However, the methodology can be used to obtain several derivatives, just by modifications in the substitution pattern of the building blocks (**60**) and (**61**).

## 5. Conclusions

This review explores the family of xanthones isolated from 2010 to 2021 and demonstrates the diversity of works carried out in this field. We verified that many xanthones were isolated from marine sources, particularly marine-derived fungi. The compounds discussed here demonstrated potential in terms of bioactive properties, such as antitumor and antibacterial. However, in our opinion, most of the compounds need to be studied more carefully, involving the use of proper positive control, statistical analysis, toxicological studies, mechanisms of action, and in vivo assays.

In recent years, xanthones have been explored as compounds with possible applications in medicine. They have been tested for different and varied bioactivities, showing promising results, which intensifies the motivation to study their structure and potential applications in treating various diseases. Given that these compounds are sometimes found in sources that are difficult to access, it is essential to find pathways that allow their synthesis without resorting to their natural origin. This review discusses the possibility of some of these marine xanthones being obtained through their total synthesis. Unfortunately, only the total syntheses of secalonic acid D and some yicathins have been reported until now. In our opinion, although some marine xanthones synthesis would be a challenge, it is crucial to develop efficient methodologies to obtain at least the more bioactive ones by total synthesis.

## Figures and Tables

**Table 1:** Summary of the biological evaluations results.

Compound	Source	Antibacterial (Positive Control Value)	Cytotoxic (Positive Control Value)
JBIR-99 (**45a**)	marine sponge-derived fungus *Tritirachium* sp. Sp081112MEf2 and marine fungus *Engyodontium album* Strain LF069	*Staphylococcus epidermidis* IC_50_ 0.21 ± 0.09 μM (chloramphenicol IC_50_ 1.8 ± 0.04 μM) *Staphylococcus aureus* IC_50_ 0.25 ± 0.07 μM (chloramphenicol IC_50_ 2.9 ± 0.5 μM)	NIH 3T3 IC_50_ 13.2 ± 2.7 μM (amoxifen citrate IC_50_ 16.5 ± 0.2 μM)
Engyodontochone B (**45b**)	marine fungus *Engyodontium album* Strain LF069	*Staphylococcus epidermidis* IC_50_ 0.22 ± 0.03 μM (chloramphenicol IC_50_ 1.8 ± 0.04 μM) *Staphylococcus aureus* IC_50_ 0.24 ± 0.04 μM (chloramphenicol IC_50_ 2.9 ± 0.5 μM)	NIH 3T3 IC_50_ 14.4 ± 2.6 μM (amoxifen citrate IC_50_ 16.5 ± 0.2 μM)
JBIR-97/98 (**46a**)	marine sponge-derived fungus *Tritirachium* sp. Sp081112MEf2 and marine fungus *Engyodontium album* Strain LF069	*Staphylococcus epidermidis* IC_50_ 0.20 ± 0.04 μM (chloramphenicol IC_50_ 1.8 ± 0.04 μM) *Staphylococcus aureus* IC_50_ 0.19 ± 0.02 μM (chloramphenicol IC_50_ 2.9 ± 0.5 μM)	NIH 3T3 IC_50_ 14.0 ± 1.1 μM (amoxifen citrate IC_50_ 16.5 ± 0.2 μM)
Engyodontochone A (**46b**)	marine fungus *Engyodontium album* Strain LF069	*Staphylococcus epidermidis* IC_50_ 0.19 ± 0.04 μM (chloramphenicol IC_50_ 1.8 ± 0.04 μM) *Staphylococcus aureus* IC_50_ 0.17 ± 0.02 μM (chloramphenicol IC_50_ 2.9 ± 0.5 μM)	NIH 3T3 IC_50_ 11.0 ± 1.6 μM (amoxifen citrate IC_50_ 16.5 ± 0.2 μM)
Compound (**8**)	marine fungus *Talaromyces islandicus* EN-501 found in red alga *Laurencia okamurai*	*Escherichia coli* MIC 32 μg/mL (chloramphenicol MIC 1 μg/mL) *Pseudomonas aeruginosa* MIC 32 μg/mL (chloramphenicol MIC 4 μg/mL) *Staphylococcus aureus* MIC > 64 μg/mL (chloramphenicol MIC 2 μg/mL) *Vibrio alginolyticus* MIC 32 μg/mL (chloramphenicol MIC 0.5 μg/mL) *Vibrio harveyi* MIC 32 μg/mL (chloramphenicol MIC 2 μg/mL) *Vibrio parahaemolyticus* MIC 32 μg/mL (chloramphenicol MIC 2 μg/mL)	
Aspergixanthone A (**20**)	marine fungus *Aspergillus* sp. ZA-01	*Micrococcus lysodeikticus* *Bacillus anthraci* *Salmonella typhi* *Enterobacter aerogenes*	MDA-MB-231 IC_50_ > 10.0 μM (cisplatin IC_50_ 1.3 μM) MCF-7 IC_50_ > 10.0 μM (cisplatin IC_50_ 0.97 μM) MGC-803 IC_50_ > 10.0 μM (cisplatin IC_50_ 1.1 μM) HeLa IC_50_ > 10.0 μM (cisplatin IC_50_ 0.82 μM) A-549 IC_50_ 1.80 μM (cisplatin IC_50_ 0.74 μM)
Aspergixanthone B (**21**)	marine fungus *Aspergillus* sp. ZA-01	*Micrococcus lysodeikticus* *Bacillus anthraci* *Salmonella typhi* *Enterobacter aerogenes*	MDA-MB-231 IC_50_ > 10.0 μM (cisplatin IC_50_ 1.3 μM) MCF-7 IC_50_ > 10.0 μM (cisplatin IC_50_ 0.97 μM) MGC-803 IC_50_ > 10.0 μM (cisplatin IC_50_ 1.1 μM) HeLa IC_50_ > 10.0 μM (cisplatin IC_50_ 0.82 μM) A-549 IC_50_ >10.0 μM (cisplatin IC_50_ 0.74 μM)
Aspergixanthone C (**22**)	marine fungus *Aspergillus* sp. ZA-01	*Micrococcus lysodeikticus* *Bacillus anthraci* *Salmonella typhi* *Enterobacter aerogenes*	MDA-MB-231 IC_50_ 3.3 μM (cisplatin IC_50_ 1.3 μM) MCF-7 IC_50_ 2.8 μM (cisplatin IC_50_ 0.97 μM) MGC-803 IC_50_ 3.6 μM (cisplatin IC_50_ 1.1 μM) HeLa IC_50_ 2.9 μM (cisplatin IC_50_ 0.82 μM) A-549 IC_50_ 3.2 μM (cisplatin IC_50_ 0.74 μM)
Aspergixanthone E (**23**)	marine fungus *Aspergillus* sp. ZA-01	*Micrococcus lysodeikticus* *Bacillus anthraci* *Salmonella typhi* *Enterobacter aerogenes*	MDA-MB-231 IC_50_ > 10.0 μM (cisplatin IC_50_ 1.3 μM) MCF-7 IC_50_ > 10.0 μM (cisplatin IC_50_ 0.97 μM) MGC-803 IC_50_ > 10.0 μM (cisplatin IC_50_ 1.1 μM) HeLa IC_50_ > 10.0 μM (cisplatin IC_50_ 0.82 μM) A-549 IC_50_ > 10.0 μM (cisplatin IC_50_ 0.74 μM)
Aspergixanthone F (**24**)	marine fungus *Aspergillus* sp. ZA-01	*Micrococcus lysodeikticus* *Bacillus anthraci* *Salmonella typhi* *Enterobacter aerogenes*	MDA-MB-231 IC_50_ 9.8 μM (cisplatin IC_50_ 1.3 μM) MCF-7 IC_50_ 2.7 μM (cisplatin IC_50_ 0.97 μM) MGC-803 IC_50_ 3.6 μM (cisplatin IC_50_ 1.1 μM) HeLa IC_50_ 1.7 μM (cisplatin IC_50_ 0.82 μM) A-549 IC_50_ 1.1 μM (cisplatin IC_50_ 0.74 μM)
Aspergixanthone D (**26**)	marine fungus *Aspergillus* sp. ZA-01	*Micrococcus lysodeikticus* *Bacillus anthraci* *Salmonella typhi* *Enterobacter aerogenes*	MDA-MB-231 IC_50_ > 10.0 μM (cisplatin IC_50_ 1.3 μM) MCF-7 IC_50_ > 10.0 μM (cisplatin IC_50_ 0.97 μM) MGC-803 IC_50_ > 10.0 μM (cisplatin IC_50_ 1.1 μM) HeLa IC_50_ > 10.0 μM (cisplatin IC_50_ 0.82 μM) A-549 IC_50_ > 10.0 μM (cisplatin IC_50_ 0.74 μM)
Aspergixanthone G (**28**)	marine fungus *Aspergillus* sp. ZA-01	*Micrococcus lysodeikticus* MIC 0.78 μg/mL *Bacillus anthraci* MIC 12.5 μg/mL *Salmonella typhi* MIC 6.13 μg/mL *Enterobacter aerogenes* MIC 6.13 μg/mL	MDA-MB-231 IC_50_ > 10.0 μM (cisplatin IC_50_ 1.3 μM) MCF-7 IC_50_ > 10.0 μM (cisplatin IC_50_ 0.97 μM) MGC-803 IC_50_ > 10.0 μM (cisplatin IC_50_ 1.1 μM) HeLa IC_50_ > 10.0 μM (cisplatin IC_50_ 0.82 μM) A-549 IC_50_ > 10.0 μM (cisplatin IC_50_ 0.74 μM)
Aspergixanthone H (**29**)	marine fungus *Aspergillus* sp. ZA-01	*Micrococcus lysodeikticus* MIC 6.13 μg/mL *Bacillus anthraci* MIC 12.5 μg/mL *Salmonella typhi* MIC 6.13 μg/mL *Enterobacter aerogenes* MIC 6.13 μg/mL	MDA-MB-231 IC_50_ > 10.0 μM (cisplatin IC_50_ 1.3 μM) MCF-7 IC_50_ > 10.0 μM (cisplatin IC_50_ 0.97 μM) MGC-803 IC_50_ > 10.0 μM (cisplatin IC_50_ 1.1 μM) HeLa IC_50_ > 10.0 μM (cisplatin IC_50_ 0.82 μM) A-549 IC_50_ > 10.0 μM (cisplatin IC_50_ 0.74 μM)
Aspergixanthone I (**27**)	marine fungus *Aspergillus* sp. ZA-01	*Vibrio anguillarum* MIC 1.56 µM (ciprofloxacin MIC 0.312 µM) *Vibrio parahemolyticus* MIC 1.56 µM (ciprofloxacin MIC 0.078 µM) *Vibrio alginolyticus* MIC 3.12 µM (ciprofloxacin MIC 0.625 µM)	
Aspergixanthone J (**30**)	marine fungus *Aspergillus* sp. ZA-01	*Vibrio anguillarum* MIC 6.25 µM (ciprofloxacin MIC 0.312 µM) *Vibrio parahemolyticus* MIC 25 µM (ciprofloxacin MIC 0.078 µM) *Vibrio alginolyticus* MIC 25 µM (ciprofloxacin MIC 0.625 µM)	
Aspergixanthone K (**31**)	marine fungus *Aspergillus* sp. ZA-01	*Vibrio anguillarum* MIC 3.12 µM (ciprofloxacin MIC 0.312 µM) *Vibrio parahemolyticus* MIC 25.0 µM (ciprofloxacin MIC 0.078 µM) *Vibrio alginolyticus* MIC 12.5 µM (ciprofloxacin MIC 0.625 µM)	
Citreamicin θ A (**32**)	marine *Streptomyces* sp.	*Staphylococcus haemolyticus* UST950701-004 MIC 0.5 μg/mL (penicillin G MIC 0.13 μg/mL, streptomycin MIC 8.0 μg/mL) *Staphylococcus aureus* UST950701-005 MIC 1.0 μg/mL (penicillin G MIC 0.25 μg/mL, streptomycin MIC 8.0 μg/mL) *Bacillus subtillis* 769 MIC 0.25 μg/mL (penicillin G MIC 0.13 μg/mL, streptomycin MIC 8.0 μg/mL) *Staphylococcus aureus* ATCC43300 MIC 0.25 μg/mL (penicillin G MIC > 50 μg/mL, streptomycin MIC > 50 μg/mL)	HeLa IC_50_ 0.055 μg/mL (cisplatin IC_50_ 18.14 μg/mL)
Citreamicin θ B (**33**)	marine *Streptomyces* sp.	*Staphylococcus haemolyticus* UST950701-004 MIC 0.5 μg/mL (penicillin G MIC 0.13 μg/mL, streptomycin MIC 8.0 μg/mL) *Staphylococcus aureus* UST950701-005 MIC 1.0 μg/mL (penicillin G MIC 0.25 μg/mL, streptomycin MIC 8.0 μg/mL) *Bacillus subtillis* 769 MIC 0.25 μg/mL (penicillin G MIC 0.13 μg/mL, streptomycin MIC 8.0 μg/mL) *Staphylococcus aureus* ATCC43300 MIC 0.25 μg/mL (penicillin G MIC > 50 μg/mL, streptomycin MIC > 50 μg/mL)	HeLa IC_50_ 0.072 μg/mL (cisplatin IC_50_ 18.14 μg/mL)
Citreaglycon A (**35**)	marine *Streptomyces* sp.	*Staphylococcus haemolyticus* UST950701-004 MIC 8.0 μg/mL (penicillin G MIC 0.13 μg/mL, streptomycin MIC 8.0 μg/mL) *Staphylococcus aureus* UST950701-005 MIC 16.0 μg/mL (penicillin G MIC 0.25 μg/mL, streptomycin MIC 8.0 μg/mL) *Bacillus subtillis* 769 MIC 8.0 μg/mL (penicillin G MIC 0.13 μg/mL, streptomycin MIC 8.0 μg/mL) *Staphylococcus aureus* ATCC43300 MIC 8.0 μg/mL (penicillin G MIC > 50 μg/mL, streptomycin MIC > 50 μg/mL)	HeLa IC_50_ > 40 μg/mL (cisplatin IC_50_ 18.14 μg/mL)
Dehydrocitreaglycon A (**36**)	marine *Streptomyces* sp.	*Staphylococcus haemolyticus* UST950701-004 MIC 8.0 μg/mL (penicillin G MIC 0.13 μg/mL, streptomycin MIC 8.0 μg/mL) *Staphylococcus aureus* UST950701-005 MIC 16.0 μg/mL (penicillin G MIC 0.25 μg/mL, streptomycin MIC 8.0 μg/mL) *Bacillus subtillis* 769 MIC > 50 μg/mL (penicillin G MIC 0.13 μg/mL, streptomycin MIC 8.0 μg/mL) *Staphylococcus aureus* ATCC43300 MIC 0.25 μg/mL (penicillin G MIC > 50 μg/mL, streptomycin MIC > 50 μg/mL)	HeLa IC_50_ > 40 μg/mL (cisplatin IC_50_ 18.14 μg/mL)
Buanmycin (**34**)	marine *Streptomyces* sp.	*Staphylococcus aureus* MIC 10.5 μΜ (ampicillin MIC 4.5 μΜ) *Bacillus subtilis* MIC 0.7 μM (ampicillin MIC 1.1 μΜ) *Kocuria rhizophila* MIC 10.5 μM (ampicillin MIC 0.6 μΜ) *Salmonella enterica* MIC 0.7 μM (ampicillin MIC 4.5 μΜ) *Proteus hauseri* MIC 21.1 μM (ampicillin MIC < 0.6 μΜ)	A-549 IC_50_ 1.7 μΜ (etoposide IC_50_ 0.8 μΜ) HCT-116 IC_50_ 0.9 μM (etoposide IC_50_ 1.9 μΜ) SNU-638 IC_50_ 0.8 μM (etoposide IC_50_ 0.5 μΜ) K-562 IC_50_ > 100 μM (etoposide IC_50_ 3.1 μΜ) SK-HEP1 IC_50_ 1.9 μM (etoposide IC_50_ 1.1 μΜ) MDA-MB-231 IC_50_ 1.2 μM (etoposide IC_50_ 10.6 μΜ)
Compound (**7**)	marine fungus *Phomopsis* sp. (No. SK7RN3G1)		Hep-2 IC_50_ 8 μg/mL HepG2 IC_50_ 9 μg/mL
Versixanthone A (**39**)	fungus *Aspergillus versicolor* HDN1009 found in soil around a mangrove		HL-60 IC_50_ 2.6 μM (doxorubicin hydrochloride IC_50_ 0.02 μM) K562 IC_50_ 7.1 μM (doxorubicin hydrochloride IC_50_ 0.3 μM) A549 IC_50_ > 50 μM (doxorubicin hydrochloride IC_50_ 0.2 μM) H1975 IC_50_ 11.2 μM (doxorubicin hydrochloride IC_50_ 0.8 μM) 803 IC_50_ > 50 μM (doxorubicin hydrochloride IC_50_ 0.2 μM) HO-8910 IC_50_ 10.1 μM (doxorubicin hydrochloride IC_50_ 0.5 μM)
Versixanthone B (**40**)	fungus *Aspergillus versicolor* HDN1009 found in soil around a mangrove		HL-60 IC_50_ 9.9 μM (doxorubicin hydrochloride IC_50_ 0.02 μM) A549 IC_50_ > 50 μM (doxorubicin hydrochloride IC_50_ 0.2 μM) H1975 IC_50_ > 50 μM (doxorubicin hydrochloride IC_50_ 0.8 μM) 803 IC_50_ 21.6 μM (doxorubicin hydrochloride IC_50_ 0.2 μM) HO-8910 IC_50_ > 50 μM (doxorubicin hydrochloride IC_50_ 0.5 μM)
Versixanthone C (**41**)	fungus *Aspergillus versicolor* HDN1009 found in soil around a mangrove		HL-60 IC_50_ 7.8 μM (doxorubicin hydrochloride IC_50_ 0.02 μM) K562 IC_50_ 18.2 μM (doxorubicin hydrochloride IC_50_ 0.3 μM) A549 IC_50_ > 50 μM (doxorubicin hydrochloride IC_50_ 0.2 μM) H1975 IC_50_ 25.6 μM (doxorubicin hydrochloride IC_50_ 0.8 μM) HO-8910 IC_50_ > 50 μM (doxorubicin hydrochloride IC_50_ 0.5 μM) HCT-116 IC_50_ > 50 μM (doxorubicin hydrochloride IC_50_ 0.2 μM)
Versixanthone D (**42**)	fungus *Aspergillus versicolor* HDN1009 found in soil around a mangrove		HL-60 IC_50_ 3.3 μM (doxorubicin hydrochloride IC_50_ 0.02 μM) K562 IC_50_ 9.1 μM (doxorubicin hydrochloride IC_50_ 0.3 μM) A549 IC_50_ 12.7 μM (doxorubicin hydrochloride IC_50_ 0.2 μM) H1975 IC_50_ > 50 μM (doxorubicin hydrochloride IC_50_ 0.8 μM) 803 IC_50_ 9.8 μM (doxorubicin hydrochloride IC_50_ 0.2 μM) HO-8910 IC_50_ 13.9 μM (doxorubicin hydrochloride IC_50_ 0.5 μM) HCT-116 IC_50_ 6.1 μM (doxorubicin hydrochloride IC_50_ 0.2 μM)
Versixanthone E (**43**)	fungus *Aspergillus versicolor* HDN1009 found in soil around a mangrove		HL-60 IC_50_ 1.6 μM (doxorubicin hydrochloride IC_50_ 0.02 μM) K562 IC_50_ 11.1 μM (doxorubicin hydrochloride IC_50_ 0.3 μM) H1975 IC_50_ 2.7 μM (doxorubicin hydrochloride IC_50_ 0.8 μM) 803 IC_50_ 2.2 μM (doxorubicin hydrochloride IC_50_ 0.2 μM) HO-8910 IC_50_ 2.0 μM (doxorubicin hydrochloride IC_50_ 0.5 μM)
Versixanthone F (**44**)	fungus *Aspergillus versicolor* HDN1009 found in soil around a mangrove		HL-60 IC_50_ 2.7 μM (doxorubicin hydrochloride IC_50_ 0.02 μM) K562 IC_50_ 6.7 μM (doxorubicin hydrochloride IC_50_ 0.3 μM) A549 IC_50_ 10.6 μM (doxorubicin hydrochloride IC_50_ 0.2 μM) HO-8910 IC_50_ 20.8 μM (doxorubicin hydrochloride IC_50_ 0.5 μM) HCT-116 IC_50_ 0.7 μM (doxorubicin hydrochloride IC_50_ 0.2 μM)
Versixanthone G (**47**)	fungus *Aspergillus versicolor* HDN1009 found in soil around a mangrove		HL-60 IC_50_ 13.4 μM K562 IC_50_ 20.9 μM A549 IC_50_ 17.8 μM H1975 IC_50_ 9.8 μM MGC803 IC_50_ 4.6 μM HEK293 IC_50_ > 50 μM HO-8910 IC_50_ 9.6 μM HCT-116 IC_50_ 16.2 μM
Versixanthone H (**48**)	fungus *Aspergillus versicolor* HDN1009 found in soil around a mangrove		HL-60 IC_50_ 6.9 μM K562 IC_50_ 22.1 μM A549 IC_50_ 19.2 μM H1975 IC_50_ 5.3 μM MGC803 IC_50_ 6.2 μM HEK293 IC_50_ > 50 μM HO-8910 IC_50_ 6.9 μM HCT-116 IC_50_ 15.2 μM
Versixanthone I (**49**)	fungus *Aspergillus versicolor* HDN1009 found in soil around a mangrove		HL-60 IC_50_ 27.8 μM K562 IC_50_ >50 μM A549 IC_50_ > 50 μM H1975 IC_50_ > 50 μM HEK293 IC_50_ > 50 μM HO-8910 IC_50_ > 50 μM HCT-116 IC_50_ > 50 μM
Versixanthone J (**50**)	fungus *Aspergillus versicolor* HDN1009 found in soil around a mangrove		HL-60 IC_50_ 47.3 μM K562 IC_50_ > 50 μM A549 IC_50_ > 50 μM H1975 IC_50_ > 50 μM HEK293 IC_50_ > 50 μM HO-8910 IC_50_ > 50 μM HCT-116 IC_50_ > 50 μM
Versixanthone K (**51**)	fungus *Aspergillus versicolor* HDN1009 found in soil around a mangrove		HL-60 IC_50_ 49.5 μM K562 IC_50_ > 50 μM A549 IC_50_ > 50 μM H1975 IC_50_ 49.5 μM MGC803 IC_50_ > 50 μM HEK293 IC_50_ > 50 μM HO-8910 IC_50_ > 50 μM HCT-116 IC_50_ > 50 μM

IC_50_ = The concentration causing 50% inhibition of cell survival; MIC = minimum inhibitory concentration; ACC-MESO-1 = human malignant pleural mesothelioma cell line; A-549, human lung cancer; BEL-7402 = human hepatocellular carcinoma cell line; C-A120 = doxorubicin-selected derivative ABCC1-overexpressing cell line; GLC-82 = human lung cancer cell line; H460 = human lung cancer cell line; H1975 = human lung cancer cell line; HCT-15, human colon cancer; HCT-116 = human colorectal carcinoma cell line; HEK-293 = human embryonic kidney cell line; HeLa = Human cervical cancer cell line; Hep-2 = Human Epidermoid carcinoma; HepG2 = Human liver cancer cell line; HL-60 = human leukemia cell line; HO-8910 = human ovarian cancer cell line; K562 = leukemia cell line; KB = human oral epidermoid carcinoma cell line; KB-3-1 = human epidermoid carcinoma cell line; KBv200 = vincristine-selected derivative ABCB1-overexpressing cell line; MCF-7 = Breast adenocarcinoma cell line; MCF-7/Adr = doxorubicin-selected derivative ABCB1-overexpressing cell line; MDA-MB-231 = human breast cancer cell line; MGC-803 = Gastric mucinous adenocarcinoma cell line; NIH-3T3 = mouse fibroblasts cell line; S1 = human colon carcinoma cell line; S1-M1-80 = mitoxantrone-selected derivative ABCG2-overexpressing cell line; SK-HEP1 = human liver cancer cell line; SK-MEL-2, human skin cancer; SK-OV-3, human ovarian cancer; SNU-638 = human gastric cancer cell line; XF- 498, human CNS cancer.
